# Nanoparticulated Anti-Programmed Cell Death-1 Antibody Improves Localized Immune Checkpoint Blockade Therapy

**DOI:** 10.34133/bmr.0221

**Published:** 2025-07-04

**Authors:** Khizra Mujahid, Muhammad Arif Aslam, Kai Han, Sejin Son, Jutaek Nam

**Affiliations:** ^1^ College of Pharmacy, Chonnam National University, Gwangju 61186, South Korea.; ^2^ State Key Laboratory of Natural Medicines, China Pharmaceutical University, Nanjing 211198, China.; ^3^ Department of Pharmaceutics, China Pharmaceutical University, Nanjing 21009, China.; ^4^ Department of Biological Sciences, Inha University, Incheon 22212, South Korea.; ^5^ Department of Biological Sciences and Bioengineering, Inha University/Industry-Academia Interactive R&E Center for Bioprocess Innovation, Inha University, Incheon, South Korea.

## Abstract

Immune checkpoint inhibitors (ICIs) have successfully transformed clinical oncology against various cancers. However, their widespread utility is limited by low response rates and severe adverse events; thus, a safe and effective approach is required to address these issues. Here, we report the nanoengineering of an anti-programmed cell death-1 antibody (aPD-1) to boost the therapeutic effects following direct local administration into tumors. Specifically, we prepared an aPD-1 nanoformulation using biocompatible mesoporous polydopamine nanoparticles (MPNs) that allow facile and efficient surface functionalization of aPD-1 via latent reactivity to proteins. The nanoformulation increased the antagonistic activity of aPD-1 against PD-1 receptors by enhancing their avidity interactions, effectively blocking PD-1 immune checkpoint signaling in T cells to restore their activation and effector function. The nanoformulation administered via local intratumoral injection enhanced tumor retention of aPD-1 and elicited strong antitumor efficacy against local tumors and long-term tumor recurrence. Our results indicate that robust immune checkpoint signaling blockade in the local tumors using nano-ICI treatment can effectively orchestrate antitumor immunity for local and systemic cancer treatment. Overall, this study underscores the potential of a biomaterial-based nanoengineering approach for improving the efficacy and safety of antibody-based ICI therapy with localized tumor treatment.

## Introduction

Cancer immunotherapy harnesses immune systems to target, recognize, and kill cancer cells, offering a new clinical cancer treatment with markedly improved patient survival compared to traditional cancer therapies [1]. In particular, T cells play an indispensable role in controlling tumor outgrowth [2]; however, the antitumor effector function of T cells is often impaired in tumors by various inhibitory receptor signaling, hampering the therapeutic outcomes [3]. Programmed cell death-1 (PD-1) is the most recognized immune checkpoint receptor expressed in T cells, which transmits suppressive signals, ultimately leading to T cell dysfunction upon binding to PD-L1 and/or PD-L2 ligands [4,5]. Importantly, most cancer cells express PD-L1 to hijack the PD-1/PD-L1 axis, thereby impairing and escaping antitumor T cell responses [6]. Blocking antibodies against the PD-1/PD-L1 axis, such as nivolumab and pembrolizumab, can reinvigorate T cells in the antitumor activity, providing clinical efficacy against multiple tumors, including melanoma, non-small cell lung cancer, and renal cell carcinoma [7,8]. However, 50% to 80% of patients fail to respond to this treatment option, and most experience immune-related adverse events, requiring an effective and safe approach for improving the performance of antibody-based immune checkpoint inhibitors (ICIs) [9,10].

Nanoengineering has been extensively investigated for drug delivery applications. Nanoparticle (NP) formulations can favorably modulate the physicochemical properties and functional activities of drug molecules for target-specific delivery and enhanced therapeutic action, increasing their on-target therapeutic efficacy and reducing off-target side effects [11,12]. Recent studies also demonstrated that nanoformulations of ICI antibodies, either displayed on the surface or entrapped inside NPs, can promote tumor accumulation, target receptor binding, and antagonistic effects of ICIs, resulting in improved therapeutic efficacy in preclinical studies [13,14]. Nonetheless, previous nano-ICI strategies mostly relied on the combined treatments of other therapies, including immune adjuvants, photothermal therapy, and cytokines, to achieve robust therapeutic efficiency [15–18]. Although promising for improving antitumor efficacy via multiple distinct or synergistic drug activities, the combination therapy often involves complex synthesis and post-modification of NPs and can exaggerate systemic toxicity and side effects, presenting technical and practical challenges. In particular, the complex fabrication processes make large-scale production costly and complicate quality control, limiting further clinical translation [19,20]. Moreover, most previous studies lack detailed mechanistic insight into how nanoformulation modulates target receptor interactions and/or antitumor immune responses of ICIs, which is important for rationale-based application of nano-ICI therapy [21–23].

**Fig. 1.**
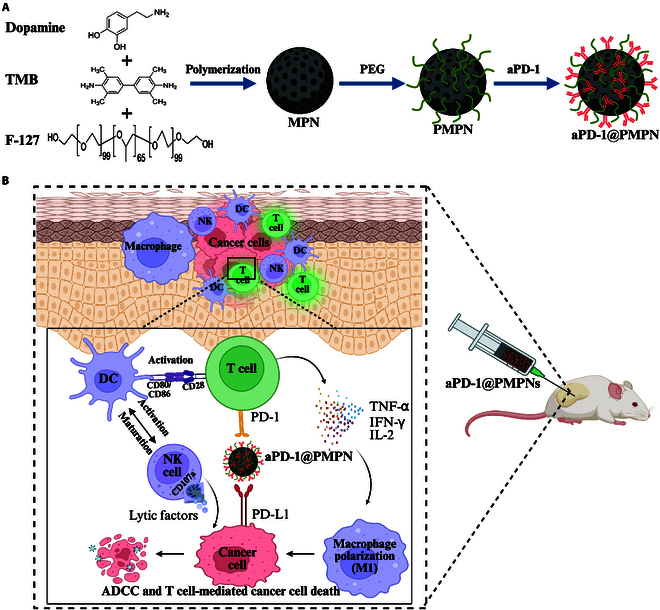
Schematic illustration of the nano-ICI therapy using an aPD-1 nanoformulation. (A) Preparation of PEGylated MPNs (PMPNs) and subsequent surface functionalization of aPD-1. (B) Antitumor immune responses following local intratumoral injection of aPD-1@PMPNs, where aPD-1@PMPNs block the PD-1 receptors in T cells to restore and orchestrate antitumor effector functions in the tumors.

Polydopamine (PDA) is a mussel-inspired biomimetic polymer that has garnered substantial interest for biological applications due to its intrinsic biocompatibility, biodegradability, simple preparation, and versatile functionalization [24]. In this study, we utilized mesoporous PDA (MPDA) NPs to nanoengineer an anti-PD-1 antibody (aPD-1) and evaluated its application in nano-ICI therapy (Fig. 1). MPDA NPs exhibit high surface area with the latent reactivity of PDA toward nucleophiles, such as amine and thiol groups, allowing facile and efficient surface functionalization of aPD-1 via simple mixing and reaction [25]. In particular, we demonstrated that the nonsaturated surface passivation of MPDA NPs with polyethylene glycol (PEG) renders the fabrication of a stable and biocompatible aPD-1 nanoformulation. NP formulations significantly potentiated T cell engagement of aPD-1 and its antagonistic effect against the PD-1 signaling axis, significantly boosting the therapeutic efficacy of ICIs as a standalone cancer treatment. Importantly, the local nano-ICI therapy via direct intratumoral administration effectively regulated local and systemic antitumor immunity, leading to strong and durable therapeutic effects against both local tumors and long-term tumor recurrence. The facile nanoengineering and localized administration of aPD-1 demonstrated in this study suggest a promising approach that can improve the efficacy and safety of antibody-based ICI therapy while also addressing the technical challenges for potential clinical applications.

## Materials and Methods

### Materials

Dopamine hydrochloride, Pluronic F-127 (F127), poly(ethylene glycol) methyl ether thiol [thiolated PEG, number-average molecular weight (Mn) 6,000], and 4′,6-diamidino-2-phenylindole (DAPI) were obtained from Sigma Aldrich. Phosphate-buffered saline (PBS), fetal bovine serum (FBS), Dulbecco’s modified Eagle’s medium (DMEM), RPMI 1640 medium, 0.25% trypsin–EDTA, and penicillin/streptomycin were obtained from Gibco. Anti-mouse PD-1 (CD279) was purchased from Bio X Cell. Alexa Fluor 488 NHS ester, Alexa Fluor 647 NHS ester, and LysoTracker Red DND 99 were obtained from Thermo Fisher Scientific. Mouse IFN-gamma DuoSet ELISA, mouse TNF-alpha DuoSet ELISA, and mouse IL-2 DuoSet ELISA were purchased from R&D Systems. The antibodies for flow cytometry analysis were obtained from eBioscience, as summarized in Table S1.

### Synthesis of MPNs, PMPNs, and aPD-1@PMPNs

Dopamine hydrochloride (0.30 g) and F127 (0.2 g) were dissolved in a mixture of deionized water (10 ml) and ethanol (10 ml) with stirring at room temperature for 30 min. Trimethyl benzene (320 μl) was added to the mixture and sonicated in a water bath for 10 min, followed by a dropwise addition of ammonia solution (750 μl) under stirring. After 2-h reaction at room temperature, mesoporous polydopamine nanoparticles (MPNs) were collected by centrifugation at 13,000*g* for 10 min and washed with a 1:1 water–ethanol mixture until the supernatant became clear. The purified MPNs were suspended in ultrapure water and reacted with thiolated PEG (10 mM) for 24 h. The PEG-modified MPNs (PMPNs) were obtained after washing with deionized water to remove unbound PEG. For aPD-1 loading, PMPNs were suspended in PBS, mixed with aPD-1, and incubated at 4 °C for 24 h, followed by purification by washing with PBS. The resulting aPD-1@PMPNs were stored in 4 °C until further use.

### NP characterization

The sizes and morphologies of the NPs were characterized using transmission electron microscopy (TEM) (JEM-2010, JEOL, Japan) and scanning electron microscopy (SEM) (JSM-7900F, JEOL, Japan). Elemental analysis was conducted using energy-dispersive x-ray spectroscopy (EDX) (JSM-7900F, JEOL, Japan). The Brunauer–Emmett–Teller (BET) surface area and pore size distribution were determined using a micromeritics instrument (ASAP 2020, Micromeritics, USA). Hydrodynamic size and zeta potential were measured using dynamic light scattering (Zetasizer Advance Pro, Malvern Panalytical, UK), and ultraviolet–visible (UV–Vis) absorbance was measured using a BioTek Synergy H1 microplate reader.

The hemolysis assay was performed by mixing 200 μl of 10% (v/v) red blood cells with 800 μl of MPNs or PMPNs in PBS at various concentrations, followed by incubation at 37 °C for 4 h. After centrifugation at 13,000*g*, the supernatant was recovered, and the absorbance was measured at 577 nm. PBS and distilled water were used as negative and positive controls, respectively. The hemolytic percentage was calculated as follows: [*A*_sample_ − *A*_PBS_]/[*A*_water_ − *A*_PBS_] × 100%, where *A*_Sample_, *A*_PBS_, and *A*_water_ are the absorbance of sample, PBS, and distilled water, respectively.

The cumulative release of aPD-1 from aPD-1@PMPNs was measured in PBS with constant stirring at 37 °C. The aliquots of the supernatant were collected at the predetermined time intervals and replaced with an equal volume of fresh PBS to maintain sink conditions. The amount of aPD-1 released at each time point was quantified using UV–Vis spectrophotometry, and the cumulative release was calculated as the percentage of initially loaded aPD-1 amount.

### Fluorophore conjugation to aPD-1

Alexa Fluor 488 NHS ester or Alexa Fluor 647 NHS ester was dissolved in dimethyl sulfoxide to a final concentration of 10 mg/ml and used immediately after reconstruction. For the conjugation, Alexa Fluor dye solution was added to an aPD-1 antibody solution dissolved in 0.1 M pH 8.3 sodium bicarbonate buffer (2 mg/ml) to generate a 1:2 antibody to dye molar ratio. After reacting at room temperature for 1 h, the Alexa-conjugated aPD-1 antibody was purified from unreacted free Alexa dye using Illustra NAP-10 desalting columns (17-0854-01, Cytiva, Marlborough, MA, USA). The final product was stored in PBS at 4 °C while protected from light.

### Isolation of OT-1 splenocytes

OT-1 splenocytes were isolated by flushing the spleen with a T cell culture medium (RPMI 1640 + 10% FBS + 1% penicillin/streptomycin + 10 mM Hepes + 1X minimum essential medium–nonessential amino acids + 1 mM sodium pyruvate + 1X 2-mercaptoethanol) while mechanically disrupting the tissue using a syringe. The cell suspension was filtered through a 70-μm cell strainer and centrifuged at 400*g* and 4 °C for 4 min. The collected cells were treated with ammonium-chloride-potassium (ACK) lysis buffer to lyse red blood cells, washed, and resuspended in a T cell culture medium.

### In vitro studies

To measure direct cytotoxicity to cancer cells, CT26 cells were cultured in 96-well plates in a culture medium (RPMI 1640 + 10% FBS + 1% penicillin/streptomycin) at the density of 2 × 10^4^ cells per well at 37 °C for 24 h. The medium was replaced with blank PBS or aPD-1, PMPNs, or aPD-1@PMPNs in PBS, and the plates were incubated for an additional 24 h. Then, the cells were washed with fresh culture medium and added to 10 μl of cell counting kit-8 (CCK-8) reagent (CK04, Dojindo, Kumamoto, Japan). After further incubation at 37 °C for 1 h, the absorbance was measured at 450 nm using a microplate reader. Results are expressed as the ratio of the absorbance to the blank PBS.

To visualize the cellular uptake of aPD-1, Alexa Fluor 488 fluorophore-conjugated aPD-1 was utilized. EL4 T cells or OT-1 splenocytes were seeded on coverslips in 24-well plates at a density of 1 × 10^5^ cells per well and cultured at 37 °C for 24 h. The cells were then treated with aPD-1 or aPD-1@PMPNs for 4 h, washed twice with PBS, and further incubated with 50 nM LysoTracker Red in PBS for 1 h. Subsequently, the cells were fixed with 4% formaldehyde for 10 min, stained with DAPI (5 μg/ml) for 10 to 15 min in the dark, and washed twice with PBS. Fluorescent images were acquired using a confocal laser scanning microscope (Carl Zeiss, Oberkochen, Germany) to analyze the cellular uptake and localization of the Alexa-conjugated aPD-1 antibody.

To measure the PD-1/PD-L1 expression and the aPD-1 binding, EL4 cells or OT-1 splenocytes were seeded in 96-well plates at 2 × 10^4^ cells or 1 × 10^5^ cells per well, respectively, and cultured at 37 °C overnight. The cells were further incubated for 4 h after the medium was replaced with phorbol 12-myristate 13-acetate (PMA)/ionomycin (for EL4 cells) or SIINFEKL peptide (for OT-1 splenocytes) for stimulation. Then, the cells were washed 3 times using PBS, recovered with 100 μl of PBS/EDTA incubated at 37 °C for 10 min, and further incubated with fixable viability dye solution (Thermo Fisher Scientific) for 30 min. Finally, cells were washed with fluorescence-activated cell sorting buffer (1% bovine serum albumin in PBS) and subjected to flow cytometry analysis (NovoCyte 3000, Agilent, USA).

To measure the proinflammatory cytokine production, the supernatants were collected after stimulation and/or sample treatments, and the concentrations of interferon-γ (IFN-γ), interleukin-2 (IL-2), and tumor necrosis factor-α (TNF-α) were measured using the respective enzyme-linked immunosorbent assay (ELISA) kits according to the manufacturer’s protocol.

### In vivo studies

Animals were cared for in accordance with the guidelines of the Institutional Animal Care and Use Committee (IACUC) of Chonnam National University (CNU), and all works conducted on animals were approved by CNU IACUC with the protocol #CNU IACUC-YB-2024-63. BALB/c mice were subcutaneously inoculated with 1 × 10^6^ CT26 colon carcinoma cells on day 0 for the therapeutic study and immune analysis. Mice were randomized into different groups and intratumorally administered either PBS, aPD-1, PMPNs, or aPD-1@PMPNs on days 11, 14, and 17. Tumor volumes and body weights were measured every 2 d. Mice were monitored for survival over 40 d. For immune analysis, mice were sacrificed on day 18, and tumor tissues, lymph nodes, spleens, and blood were harvested. Flow cytometry was performed to analyze innate and adaptive immune cells, and an ELISA assay to analyze cytokines.

For in vivo whole-body imaging, mice were intratumorally injected with MPNs, PMPNs, aPD-1, or aPD-1@PMPNs with Alexa Fluor 647 labeled on the NPs or aPD-1. Fluorescence imaging was performed in vivo using a whole-body imaging system (FOBI, CELLGENTEK Co. Ltd., South Korea) at the predefined time points indicated in the results. Fluorescence intensity was quantified using the built-in software.

### Gene expression analysis

Total RNA was extracted from target cells using TRI Reagent (MRC, Cincinnati, OH, USA). cDNA synthesis was performed using the TOPscript RT DryMIX (Enzynomics, Daejeon, South Korea). mRNA levels were measured in a 10-μl reaction volume consisting of cDNA transcripts, primer pairs, and the TOPreal SYBR Green PCR kit (Enzynomics) by real-time polymerase chain reaction (PCR) using a Bio-Rad CFX96 Real-Time PCR Detection System (Bio-Rad, Hercules, CA, USA). Gene expression levels were normalized to the *18S rRNA* levels. Primer pairs for IFN-γ, TNF-α, and 18S were used, with their forward and reverse sequences specified in Table S2.

### Western blot analysis

Target cells were lysed using radioimmunoprecipitation assay (RIPA) buffer containing protease and phosphatase inhibitors. The bicinchoninic acid (BCA) assay was employed to determine the protein concentration. Equal protein concentrations were separated using sodium dodecyl sulfate–polyacrylamide gel electrophoresis and transferred to polyvinylidene difluoride membranes. Then, the membranes were blocked with 5% nonfat dry milk in tris-buffered saline with Tween-20 (TBS-T) and incubated with primary antibodies against total and phosphorylated AKT and extracellular signal-regulated kinase (ERK) (Cell Signaling Technology) at 4 °C overnight, followed by washing and further incubation with horseradish peroxidase-conjugated secondary antibodies at room temperature for 1 h. Protein bands were visualized using an enhanced chemiluminescence detection system and quantified by densitometry (LAS-4000, Fuji Photo Film, Tokyo, Japan). The used antibodies are listed in Table S3.

### Hematoxylin and eosin staining

Paraffin-embedded sections of heart, liver, spleen, and lung were deparaffinized in xylene (3 washes, 4 min each), followed by rehydration through a graded ethanol series: 100% ethanol (twice, 3 min), 95% ethanol (3 min), and 70% ethanol (3 min). Sections were stained with hematoxylin for 1.5 min, rinsed under running tap water for 5 min, and counterstained with eosin for 2 min, followed by a brief rinse. Slides were then dehydrated in 95% ethanol (twice, 1 min) and 100% ethanol (twice, 1 min), cleared in xylene (3 washes, 2 min), and mounted with coverslips. Histological assessments were performed using an LSM 900 confocal microscope in brightfield mode at 20X magnification, and images were captured for each group and organ.

### Statistical analysis

The results are expressed as the mean ± standard error of the mean (SEM). Experiments were repeated multiple times, and each figure shows a complete dataset from a representative independent experiment. To analyze statistical differences among groups, 1- or 2-way analysis of variance (ANOVA), followed by Tukey’s multiple comparisons post hoc test, was performed using GraphPad Prism 10.2.1 (GraphPad Software).

## Results

### Synthesis and characterization of MPNs, PMPNs, and aPD-1@PMPNs

**Fig. 2.**
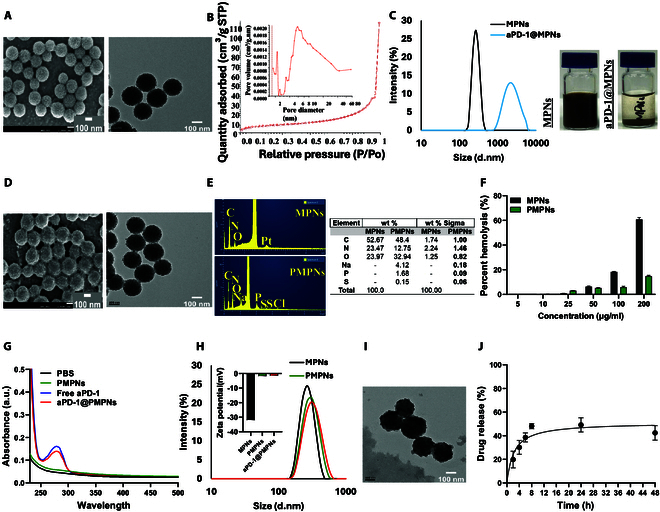
Synthesis and characterization of MPNs, PMPNs, and aPD-1@PMPNs. (A) SEM and TEM images and (B) nitrogen adsorption–desorption isotherm of MPNs. (C) Hydrodynamic sizes and photographs of MPNs and aPD-1@MPNs in PBS. (D) SEM and TEM images of PMPNs. (E) EDX analysis and (F) hemolysis assay of MPNs and PMPNs. (G) Absorption spectra and (H) hydrodynamic size and zeta potential showing successful preparation of aPD-1@PMPNs. (I) TEM image and (J) cumulative release of aPD-1 from aPD-1@PMPNs over time.

MPNs were prepared by a surfactant-templated polymerization of dopamine as described before [26] (Fig. 1A). MPNs exhibited spherical morphology and rough surface, and the average size was 188 ± 12 nm, as measured using TEM and SEM (Fig. 2A). Nitrogen adsorption–desorption analysis verified the mesoporous structure with an average pore size of 2 to 10 nm (Fig. 2B). PDA allows facile surface functionalization of proteins in a simple one-pot mixture through its latent reactivity toward amine and thiol groups while preserving inherent biological functionality of the immobilized proteins [27]. Accordingly, we first attempted to incorporate aPD-1 onto MPNs by mixing them in PBS; however, this process induced massive aggregation and precipitation, suggesting that the MPNs became unstable following surface tethering of aPD-1 (Fig. 2C). We sought to improve the colloidal stability of MPNs via surface passivation with PEG, for which thiolated PEG was employed to utilize the reactivity of thiol moiety to the PDA [25]. The resulting PEGylated MPNs (PMPNs) exhibited similar spherical morphology and mesoporous structure to MPNs, with sticky organic layers identified under SEM (Fig. 2D). Further elemental analysis demonstrated the presence of sulfur, and therefore successful PEG incorporation via sulfide linkage (Fig. 2E). We also tested and verified PEG passivation for its anti-fouling effect that improved biocompatibility of MPNs against hemolysis activity (Fig. 2F). Importantly, surface-tethered PEG did not completely block the subsequent incorporation of aPD-1. The absorption spectrum of aPD-1-loaded PMPNs (aPD-1@PMPNs) demonstrated substantial loading of aPD-1 (Fig. 2G), and the loading efficiency was calculated as ~31% based on the absorbance of free unbound aPD-1. This suggests that our protocol results in unsaturated PEG coverage on MPNs, allowing substantial room for further surface functionalization with aPD-1. We further conducted dynamic light scattering measurements to investigate the physicochemical changes of MPNs after successive surface modifications (Fig. 2H). MPNs exhibited a hydrodynamic size of 262 ± 4.6 nm and zeta potential of −32 mV, where the negative surface charge indicates the deprotonation of the phenolic groups that renders stable dispersion in aqueous solution [28,29]. The hydrodynamic size of MPNs increased to 289 ± 6.02 nm after PEG passivation, while the zeta potential changed to nearly neutral −1.73 mV. Subsequent aPD-1 loading further changed the hydrodynamic size to 305 ± 3 nm, showing a ~15-nm increase attributed to the adsorbed aPD-1, whereas the zeta potential remained unchanged at the neutral value. These results demonstrate successful PEG and aPD-1 modifications while maintaining the colloidal stability of MPNs by the outmost PEG layers. PMPNs exhibited smoothened surface after aPD-1 incorporation with a hint of organic layers (Fig. 2I). The release study showed ~40% release of aPD-1 within the first 8 h, followed by stable levels over 48 h, leaving a substantial amount of aPD-1 remaining bound to PMPNs over extended periods (Fig. 2J). Overall, we successfully prepared aPD-1@PMPNs, in which PEG plays an important role in the preparation of stable and biocompatible aPD-1 nanoformulation.

### aPD-1@PMPNs enhance T cell engagement and immune checkpoint blockade of aPD-1

**Fig. 3.**
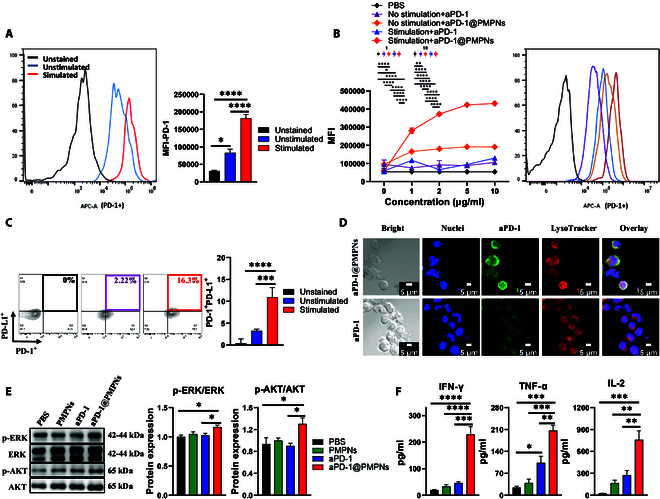
In vitro evaluation of aPD-1-based immune checkpoint blockade using EL4 T cells. (A) PD-1 expression levels measured with and without PMA/ionomycin stimulation. Flow cytometry analysis of (B) dose-dependent aPD-1 binding and (C) PD-1 and PD-L1 coexpression with or without PMA/ionomycin stimulation. (D) Confocal microscope images. (E) Western blot analysis of p-ERK, ERK, p-AKT, and AKT, and (F) the secretion of IFN-γ, TNF-α, and IL-2. The data are presented as the mean ± SEM, with *n* = 6 for (A), *n* = 3 for (B), *n* = 10 for (C), *n* = 6 for (E), and *n* = 3 for (F). *P < 0.05, **P < 0.01, ***P < 0.001, ****P < 0.0001, analyzed by using one-way ANOVA with Tukey’s multiple comparisons test.

Next, we conducted in vitro studies using EL4 murine T lymphoblast cells to evaluate the impact of immune checkpoint blockade on T cells. We first confirmed that EL4 cells express PD-1 receptors, and the expression level increases after PMA/ionomycin stimulation (Fig. 3A). This aligns with the native expression and activation-induced up-regulation of PD-1 by T cells [30]. To quantitatively assess PD-1 receptor binding, aPD-1 was covalently labeled with Alexa Fluor 488 fluorophore and incubated with EL4 cells in the form of either free aPD-1 or aPD-1@PMPNs at identical doses of aPD-1. Flow cytometry analysis of EL4 cells showed a dose-dependent increase in the mean fluorescence intensity (MFI) of aPD-1 for aPD-1@PMPNs, while free aPD-1 exhibited negligible changes in MFI regardless of the doses (Fig. 3B). Quantitatively, aPD-1@PMPNs significantly increased aPD-1 binding by 1.8-fold compared with free aPD-1 at the saturation concentration, and the difference was further amplified to 3.3-fold when samples were incubated with EL4 cells after PMA/ionomycin stimulation. These results demonstrate significantly enhanced T cell engagement of aPD-1@PMPNs in accordance with PD-1 expression level, which suggests the beneficial role of nanoformulation for improving the avidity of aPD-1 toward the PD-1 receptor to promote PD-1/aPD-1 interactions. Intriguingly, flow cytometry analysis also revealed that EL4 cells coexpress PD-1 and PD-L1, and the coexpression level was significantly increased after PMA/ionomycin stimulation, indicating dynamic regulation of both PD-1 and PD-L1 in response to T cell activation [31] (Fig. 3C). This suggests that PD-1 and PD-L1 can interact in trans between EL4 cells when presented nearby at high concentrations. Thus, this can offer an in vitro single-cell line model that can be applied to investigate the effects of immune checkpoint blockade. We conducted subsequent studies using EL4 cells pretreated with PMA/ionomycin to reflect the immune checkpoint signaling in the activated T cells. Confocal microscopy directly visualized the enhanced cellular binding and uptake of aPD-1 by the aPD-1@PMPN formulation (Fig. 3D). Although the fluorescence was mostly confined to the cell surface, some signals were also found in the cells that overlapped with lysotracker, indicating PD-1 receptor-mediated endocytosis that can potentially enhance the cytolytic potential of immune checkpoint blockade [32]. We next investigated the impact of aPD-1 engagement on the PD-1 signaling and activation of T cells. We performed Western blot analysis and observed that aPD-1@PMPN treatment significantly increased ERK and AKT phosphorylation in EL4 cells, the key downstream T cell activation events inhibited by PD-1 receptor ligation (Fig. 3E) [33]. In contrast, neither PMPNs nor free aPD-1 showed a noticeable difference compared with the PBS control. This result demonstrated that the inhibitory signaling of the PD-1 receptor is being operated in a steady state between activated EL4 cells in our experimental condition, possibly via the ligation of concomitantly up-regulated PD-L1, and aPD-1@PMPNs effectively blocked the inhibitory signaling with a strong antagonistic binding to PD-1 receptors. These findings also underscore the significance of the aPD-1 nanoformulation as individual components, including PMPNs and free aPD-1, failed to show efficacy. In addition, blocking PD-1 signaling was directly linked to the functional activation of T cells, characterized by the production of immune stimulatory proinflammatory cytokines, including IFN-γ, TNF-α, and IL-2 (Fig. 3F). Elevated cytokine secretion was observed in the EL4 cells treated with aPD-1@PMPNs, but not with other samples, consistent with the restoration of the ERK and AKT signaling cascades.

**Fig. 4.**
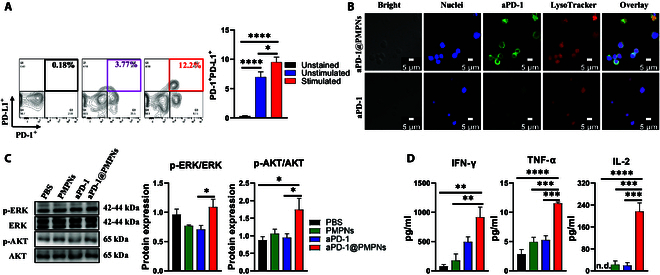
In vitro evaluation of aPD-1-based immune checkpoint blockade using OT-1 splenocytes. (A) PD-1 and PD-L1 coexpression with or without SIINFEKL peptide stimulation. (B) Confocal microscope images. (C) Western blot analysis of p-ERK, ERK, p-AKT, and AKT, and (D) the secretion of IFN-γ, TNF-α, and IL-2. The data are presented as the mean ± SEM, with *n* = 10 for (A), *n* = 6 for (C), and *n* = 3 for (D). *P < 0.05, **P < 0.01, ***P < 0.001, ****P < 0.0001, analyzed by one-way ANOVA with Tukey’s multiple comparisons test.

Next, we sought to validate the performance of aPD-1@PMPNs using pharmaceutically more relevant primary T cells. We repeated the in vitro experiments using the splenocytes obtained from OT-1 mice, a transgenic T cell receptor strain widely adapted for monitoring antigen-specific T cell responses [34]. OT-1 splenocytes with CD8^+^ T cells specific to ovalbumin peptide of amino acids 257 to 264 (SIINFEKL) were primed with the SIINFEKL peptide to simulate the clinical scenario in which T cells infiltrate tumors and recognize cancer cells; however, they become dysfunctional by PD-1 receptor ligation of PD-L1 expressed by cancer cells. SIINFEKL stimulation elevated the expression levels of both PD-1 and PD-L1 in OT-1 splenocytes; this reassembled those observed in PMA/ionomycin-stimulated EL4 cells, thus suggesting that concurrent up-regulation of PD-1 and PD-L1 is a common event in activated T cells (Fig. 4A). Subsequent experiments using SIINFEKL-stimulated OT-1 splenocytes reproduced the results of the EL4 cells, showing that aPD-1@PMPNs enhance cell binding and uptake, increase phosphorylation of ERK and AKT, and elevate proinflammatory cytokine production by OT-1 splenocytes, which confirms the general performance and utility of aPD-1@PMPNs for PD-1-targeting ICI therapy (Fig. 4B to D and Fig. S1). These results underline the potential of aPD-1@PMPN-based nano-ICI therapy for enhancing PD-1 engagement by increasing avidity interactions, thereby exerting a robust antagonistic effect against the PD-1 signaling axis to reinvigorate and promote the effector function of T cells.

### aPD-1@PMPNs exert strong antitumor efficacy

**Fig. 5.**
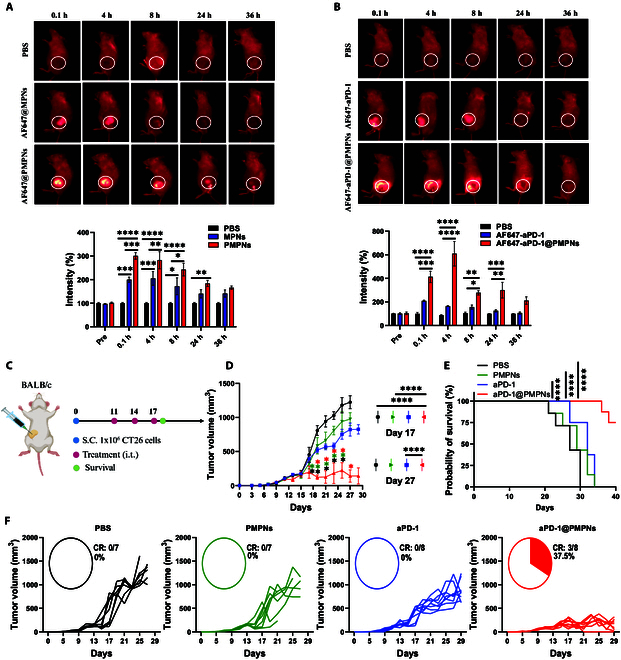
In vivo evaluation of aPD-1-based immune checkpoint blockade therapy against CT26 tumors. (A and B) In vivo whole-body fluorescence imaging for the analysis of local tumor retention. (C) Schematic illustration of the treatment regimen. (D) Average tumor growth and (F) individual tumor growth curves with the portion of complete regression. (E) Overall Kaplan–Meier survival curves of tumor-bearing mice. The data represent the mean ± SEM, with *n* = 3 for (A) and (B) and *n* = 7 (PBS, PMPNs) or 8 (aPD-1, aPD-1@PMPNs) for (D) to (F). *P < 0.05, **P < 0.01, ***P < 0.001, ****P < 0.0001, analyzed by 2-way ANOVA with Tukey’s multiple comparisons tests (A, B, and D) or log-rank (Mantel–Cox) test (E).

Having demonstrated PD-1 signaling blockade in vitro, we next investigated the in vivo therapeutic efficiency of immune checkpoint blockade using the CT26 colorectal carcinoma model. We first assessed the impact of PEG passivation on the local tumor retention of MPNs. BALB/c mice were inoculated with 1 × 10^6^ CT26 cells subcutaneously on day 0 and, when the tumors reached ~100 mm^3^ on day 11, administered intratumorally with either MPNs or PMPNs labeled with Alexa Fluor 647 for the fluorescence-based tracking of their tumor retention. PBS was used as a negative control. In vivo whole-body fluorescence imaging demonstrated enhanced local tumor retention of PMPNs compared with MPNs over 24 h (Fig. 5A). This suggests that stable single-particle dispersion via partial PEG coverage can improve tumor residence of MPNs, possibly by increasing a steric hindrance against free diffusion or because the individually dispersed particles expose high surface area for physical or chemical surface interaction with local cues that could slower particle spread in the tumor microenvironment. In addition, PMPNs also enhanced the local retention of surface-tethered aPD-1 in the tumors as quantified using Alexa Fluor 647-conjugated aPD-1 (Fig. 5B). aPD-1@PMPNs effectively retained aPD-1 in the tumors, with the local aPD-1 concentrations remaining much greater than free aPD-1 for extended periods. We also noted that this could be attributed to the enhanced engagement of aPD-1@PMPNs to PD-1-expressing target cells in the tumor, as demonstrated in vitro using PD-1-expressing T cells. Next, we investigated whether the increased tumor retention of aPD-1 could translate to improved anticancer efficacy. To evaluate the therapeutic efficacy, we established the CT26 tumor model as above and administered PMPNs, aPD-1, or aPD-1@PMPNs on days 11, 14, and 17 at an equivalent aPD-1 dose (Fig. 5C). PBS was also employed as a control group. Tumor growth was significantly suppressed in aPD-1@PMPN-treated mice, whereas mice that received other samples showed only marginal alterations, compared with the PBS control (Fig. 5D). As a result, aPD-1@PMPNs treatment led to complete tumor regression in 3 of 8 mice and markedly extended animal survival, while other treatments achieved neither complete tumor regression nor substantial extension in the survival (Fig. 5E and F). We did not observe an adverse impact on the body weight, a sign of systemic toxicity, by any samples throughout the monitoring periods (Fig. S2). These results demonstrated that aPD-1@PMPNs can improve tumor retention and antitumor efficacy of aPD-1 after local administration without causing severe systemic toxicity.

### Immunological impact of aPD-1@PMPNs on the local tumors

**Fig. 6.**
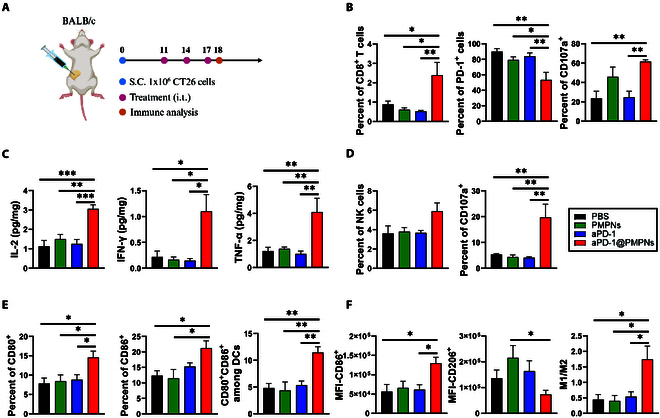
Immune analysis in the local tumors. (A) Schematics of treatment regimen. Analysis of (B) CD8^+^ T cells, (C) proinflammatory cytokines, (D) NK cells, (E) DCs, and (F) macrophages. The data are presented as the mean ± SEM, with *n* = 6 for (C) and *n* = 8 for (B) and (D) to (F). *P < 0.05, **P < 0.01, ***P < 0.001, ****P < 0.0001, analyzed by one-way ANOVA with Tukey’s multiple comparisons test.

aPD-1@PMPNs did not directly induce noticeable cytotoxicity to CT26 cells (Fig. S3), which is in line with the biocompatibility of PMPNs. Together with the strong PD-1 antagonistic activity demonstrated in vitro, this suggests that the PD-1 signaling blockade elicits robust local antitumor immune responses associated with antitumor efficacy. To interrogate the antitumor immune responses, we established CT26 tumors and administered samples as in Fig. 5C, and the tumors were excised on day 18 for the analysis of innate and adaptive immune compartments (Fig. 6A and Fig. S4). The ex vivo tumor tissues exhibited significantly decreased sizes and weight only with aPD-1@PMPN treatment (Fig. S5), which is consistent with the trend of tumor growth inhibition in Fig. 5D. Immune cell analysis revealed that aPD-1@PMPNs reduced the level of accessible PD-1 in CD8^+^ T cells, suggesting their effective engagement and occupation of PD-1 receptors (Fig. 6B) [35]. In addition, aPD-1@PMPNs increased the frequency of CD8^+^ T cells in the tumors and induced their activation characterized by up-regulated CD107a, a classical marker of degranulation (Fig. 6B and Fig. S6) [36]. Furthermore, aPD-1@PMPNs elevated the local concentrations of proinflammatory cytokines produced by activated T cells, including IL-2, IFN-γ, and TNF-α (Fig. 6C and Fig. S7). In contrast, PMPNs or free aPD-1 did not alter the accessible PD-1 receptor level, CD8^+^ T cell frequency and activity, and cytokine concentrations in the local tumors, compared with PBS control. These results demonstrate robust tumor infiltration, activation, and effector immune functions of CD8^+^ T cells triggered by aPD-1@PMPNs. It was also found that aPD-1@PMPNs promoted the infiltration of natural killer (NK) cells and their activation as shown by CD107a, activation and maturation of dendritic cells (DCs) by CD80 and CD86 costimulatory markers, and macrophage phenotype shift from anti-inflammatory M2 toward proinflammatory M1 by CD86 and CD206 as M1 and M2 markers, respectively (Fig. 6D to F and Figs. S8 to S10). These results demonstrate the favorable changes in the innate arm of antitumor immunity, which also plays pivotal roles in mounting robust antitumor immune responses and efficacy [37]. These immune cells can play complementary roles in tumor clearance, where M1 macrophages and DCs can promote the priming of CD8^+^ T cells and NK cells with antigen presentation and proinflammatory responses, and CD8^+^ T cells and NK cells directly target and kill cancer cells in an antigen-specific and nonspecific manner, respectively [38]. Such collective responses can achieve efficient tumor control with synergistic antitumor immune actions. Again, these changes in the local immune cells were observed only with aPD-1@PMPNs but not with other samples, indicating a causal relationship with the PD-1 blockade. Overall, aPD-1@PMPNs elicited robust adaptive and innate immune responses by CD8^+^ T cells, NK cells, DCs, and macrophages likely associated with the PD-1 blockade in the local tumors, which can altogether orchestrate effective antitumor immune responses, leading to strong antitumor efficacy.

### Systemic immune activation by aPD-1@PMPNs

**Fig. 7.**
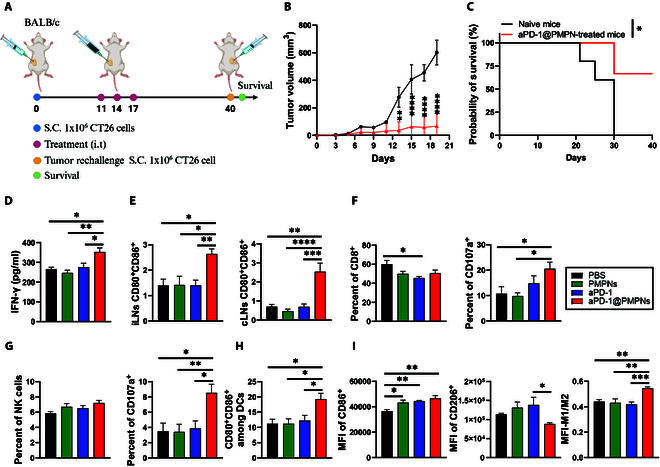
Analysis of systemic immune activation. (A) Schematics of the treatment regimen. (B) Average tumor growth and (C) overall Kaplan–Meier survival curves for the tumor rechallenge study. (D) IFN-γ level in the serum. (E) DCs in tumor-draining inguinal lymph nodes (iLNs) and non-tumor-draining contralateral lymph nodes (cLNs). Analysis of spleen for (F) CD8^+^ T cells, (G) NK cells, (H) DCs, and (I) macrophages. The data are presented as the mean ± SEM, with *n* = 3 (aPD-1@PMPN-treated mice) or 5 (naïve mice) for (B) and (C) and *n* = 6 for (D) to (I). *P < 0.05, **P < 0.01, ***P < 0.001, ****P < 0.0001, analyzed by one-way ANOVA with Tukey’s multiple comparisons test.

We also investigated the induction of systemic antitumor immunity in light that local antitumor immune activation can be spread to systemic immune responses [39]. We rechallenged the survivors previously treated with aPD-1@PMPNs with subcutaneous injection of 1 × 10^6^ CT26 cells on the contralateral flank on day 40 (Fig. 7A). We observed that 67% of mice previously treated with aPD-1@PMPNs rejected tumor rechallenge of CT26 cells, while all native mice succumbed to the tumor by day 30, indicating systemic immunological memory response for long-term protection against tumor recurrence (Fig. 7B and C). Given this result, we further analyzed the immune profiles in major peripheral organs that coordinate systemic immunity, using the same CT26 tumor model and treatment schedule as in Fig. 6A. aPD-1@PMPNs significantly elevated the concentration of IFN-γ in the serum, indicating a proinflammatory environment fostered beyond the local tumors (Fig. 7D) [40]. Despite the significant production in the tumors (Fig. 6C), IL-2 and TNF-α were not detected in the serum, possibly due to their low detection limit with serum dilution or short half-life in the systemic circulation (data not shown). In addition, aPD-1@PMPNs promoted the activation of DCs in both tumor-draining and non-tumor-draining lymph nodes, as demonstrated by the increased CD80^+^CD86^+^ double-positive population (Fig. 7E and Fig. S11). Lymph nodes serve as an activation hub for T cells as DCs can capture tumor antigens, traffic to lymph nodes, and present the tumor antigens to T cells for their priming and clonal expansion [41,42]. This process can promote antitumor immune responses of systemic T cells, which can further boost adaptive immunity against local and disseminated tumors [43]. Immune cell analysis in the spleen showed that aPD-1@PMPNs induced CD107a up-regulation by CD8^+^ T cells and NK cells, indicating their activation (Fig. 7F and G and Figs. S12 and S13). Splenic DCs and macrophages also exhibited antitumor immune phenotypes with the up-regulation of the markers for DC activation (CD80^+^CD86^+^) and M2-to-M1 macrophage polarization (CD206-to-CD86) after aPD-1@PMPN treatment (Fig. 7H and I and Figs. S14 and S15). The observed activation of CD8^+^ T cells and NK cells, alongside the activation of DCs and M1 polarization of macrophages, further emphasizes the broad reprogramming of systemic immunity toward effective antitumor immune responses [38]. In contrast, compared with the PBS control, other samples did not show noticeable changes in the cytokine or immune cell profiles in the peripheral organs. These results mirrored the local immune responses in the tumors, suggesting a link between local and systemic immune regulation. Collectively, these findings highlight that aPD-1@PMPNs favorably modulate antitumor immunity in local and systemic compartments, providing a robust therapeutic benefit against both local tumors and systemic tumor spread.

### Assessment of systemic toxicity to normal organs

**Fig. 8.**
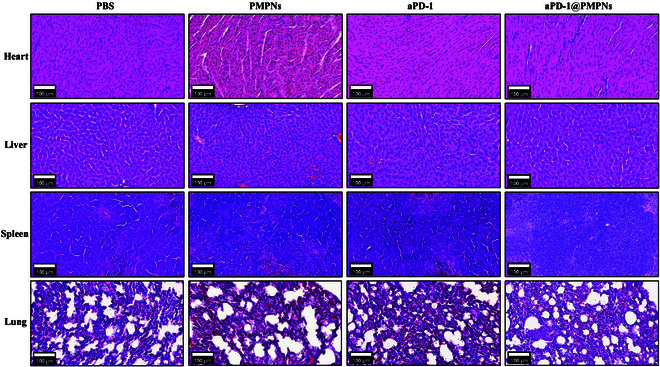
Representative H&E-stained section images of heart, liver, spleen, and lung from mice treated with PBS, PMPNs, aPD-1, or aPD-1@PMPNs. Scale bar, 100 μm.

Given that the local administration could leak to systemic circulation to result in nonspecific distribution in normal organs, and the strong immune activation could cause side effects associated with overt immune responses, we evaluated potential toxicity to the major organs including heart, liver, spleen, and lung. Histological analysis with hematoxylin and eosin (H&E) staining revealed preserved tissue architecture in all examined organs, with no signs of abnormal inflammatory cell infiltration and necrosis across PBS, PMPN, aPD-1, and aPD-1@PMPN samples (Fig. 8). This finding indicates that local intratumoral administration of biocompatible aPD-1@PMPNs does not induce detectable off-target systemic toxicity that manifests tissue damage and pathological alternation in the normal organs.

## Discussion and Conclusion

In this study, we utilized biomimetic PDA-based MPNs for the nanofabrication of aPD-1, taking advantage of their biocompatibility, biodegradability, large surface area, and facile functionalization for efficient loading and delivery of proteins [24,44,45]. We further improved the colloidal stability and biocompatibility of MPNs with unsaturated surface passivation of PEG, which allowed the construction of stable and biocompatible aPD-1 nanoformulation. Studies conducted in vitro demonstrated that aPD-1@PMPNs increased the avidity interaction of aPD-1 toward PD-1 receptors in T cells and effectively blocked their inhibitory signaling to restore the activation and effector function of T cells. Interestingly, we observed some extent of cellular internalization and localization of aPD-1@PMPNs within lysosomal compartments, suggesting PD-1 receptor-mediated endocytosis. While immune checkpoint blockade is usually considered to function at the cell surface membrane, our findings and emerging evidence [32] suggest that PD-1 receptor-mediated endocytosis of aPD-1 can contribute to the sustained receptor occupancy and checkpoint signaling inhibition to further enhance T cell activation. This may also influence intracellular signaling cascades. Mechanistically, aPD-1@PMPNs modulated PD-1 signaling pathways associated with the phosphorylation of ERK and AKT to increase the levels of p-ERK and p-AKT involved in the T cell activation. In addition, enhanced PD-1 signaling blockade reflected the improved in vivo antitumor efficacy of aPD-1@PMPNs. Following intratumoral administration, aPD-1@PMPNs extended the tumor retention of aPD-1 and subsequently exerted robust therapeutic effects against local tumors with the production of proinflammatory cytokines and the recruitment and activation of CD8^+^ T cells as well as NK cells, macrophages, and DCs. These results demonstrated that aPD-1@PMPNs orchestrate innate and adaptive immune networks to foster the local immune landscape in favor of robust antitumor immune responses and antitumor efficacy. Importantly, aPD-1@PMPNs also promoted systemic antitumor immunity, highlighting their utility for effective cancer immunotherapy beyond local tumor treatments. The rejection of the tumor rechallenge in the mice pretreated with aPD-1@PMPNs confirmed the establishment of long-term systemic immunological memory, an important indicator of the clinical responses against tumor recurrence and metastasis [46]. The immune profiles in the peripheral lymphoid organs, including the elevated proinflammatory cytokine level in the blood, activation of DCs in lymph nodes, and activation and functional maturation of CD8^+^ T cells, NK cells, macrophages, and DCs in the spleen, mirrored the local immune responses in the tumors, suggesting systemic reach of local immunological impact. Nanoengineering strategy has been widely exploited for drug delivery applications including ICI therapy; however, previous nano-ICI formulations usually exhibited limited therapeutic efficacy as a monotherapy, requiring combination approach with immune adjuvant, photothermal therapy, and cytokines for improving therapeutic outcome [15–18]. They also often involve complicated synthesis and post-modification steps, posing technical challenge in scalable manufacturing and quality control for clinical applications. In our system, PDA chemistry allows facile NP synthesis and surface functionalization, which can be beneficial for clinical-scale production of nano-ICI formulation. The robust local and systemic immune modulation and antitumor efficacy as a standalone treatment also presents a major advancement of our aPD-1@PMPN formulation over the previous ones. Systemic administration remains a dominant route for clinical ICI therapy, requiring multifaceted and sophisticated drug designs to address issues, such as rapid systemic clearance, off-target distribution, and limited tumor penetration [21,23,47]. The robust and durable systemic antitumor immune responses by local intratumoral administration suggest a promising cancer immunotherapy approach that can bypass the issues associated with systemic drug delivery while also minimizing systemic drug exposure and toxicity [48]. Despite the promise of localized immunotherapy, its reliance on the accessible tumors inherently limits broad clinical applicability against early-stage and/or deep-seated tumors. The versatility of our PDA-based platform may allow further engineering for ligand-mediated targeting, biomimetic surface modifications, or immune cell–based delivery systems to enhance circulation time, tumor homing, and therapeutic efficacy with clinically more preferred systemic administration [49]. Collectively, these findings demonstrate that robust immune checkpoint signaling blockade in the local tumors using nano-ICI treatment can elicit both local and systemic antitumor immunity to exert strong and durable therapeutic effects against advanced tumors with systemic tumor spread and recurrence.

In conclusion, we report the nanoengineering of aPD-1 using biocompatible PMPNs that allow efficient loading of proteins via simple mixing and reaction. The nanoformulation increased the binding avidity of aPD-1 toward PD-1 receptors to efficiently block the PD-1 signaling axis and subsequently promote the activation and effector function of T cells. The strong PD-1 antagonistic activity orchestrated robust antitumor immune responses not only in the local tumors but also in the systemic immune compartments, exerting remarkable therapeutic effects against primary tumors and long-term tumor recurrence. Our results highlight the potential of a biomaterial-based nanoengineering approach to improve the efficacy and safety of antibody-based ICI therapy.

## Data Availability

All data generated during the study are presented in the manuscript and the Supplementary Materials.
