# Supplementary material for: Nanoparticulated Anti-Programmed Cell Death-1 Antibody Improves Localized Immune Checkpoint Blockade Therapy
Source: Biomater Res. 2025 Jul 4;29:0221. doi: 10.34133/bmr.0221 (PMC12231238; doi:10.34133/bmr.0221)
Supplement: Supplementary 1 — Figs. S1 to S15 Tables S1 to S3 [file bmr.0221.f1.docx]

**Supplementary information**

**Nanoparticulated anti-programmed cell death-1 antibody improves localized immune checkpoint blockade therapy**

Khizra Mujahid1†, Muhammad Arif Aslam^1^†, Kai Han^2,3,4^, Sejin Son^5,6^*, Jutaek Nam1*

*1College of Pharmacy, Chonnam National University, Gwangju, 61186, South Korea*

*^2^State Key Laboratory of Natural Medicines, China Pharmaceutical University, No. 639 Longmian Dadao, Nanjing 211198, China.*

*^3^Department of Pharmaceutics, China Pharmaceutical University, Nanjing 21009, China.*

*^4^Jiangsu Key Laboratory of Drug Design and Optimization, China Pharmaceutical University, Nanjing 21009, China.*

*^5^Department of Biological Sciences, Inha University, Incheon 22212, South Korea*

*^6^Department of Biological Sciences and Bioengineering, Inha University/Industry-Academia Interactive R&E Center for Bioprocess Innovation, Inha University, Incheon, South Korea*

*^*^Co-corresponding authors: ssejin@inha.ac.kr and namj@jnu.ac.kr*

† Khizra Mujahid and Muhammad Arif Aslam contributed equally.

**
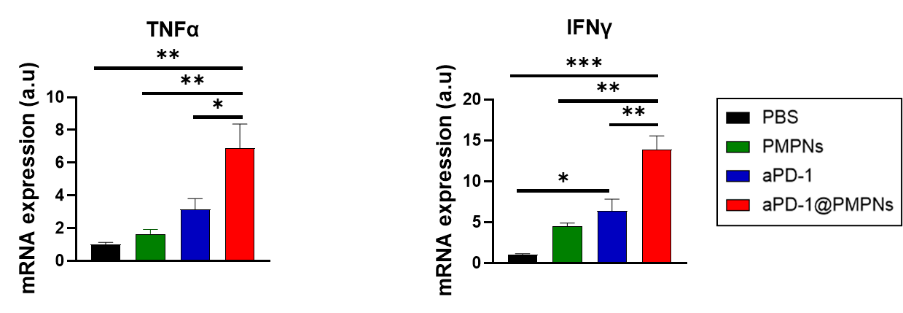
**

**Fig. 1.** Cytokine expression levels in OT-1 splenocytes.

**Fig. 2.** Body weight changes over time post sample treatment.

**Fig. 3.** Dose-dependent viability of CT26 cells.


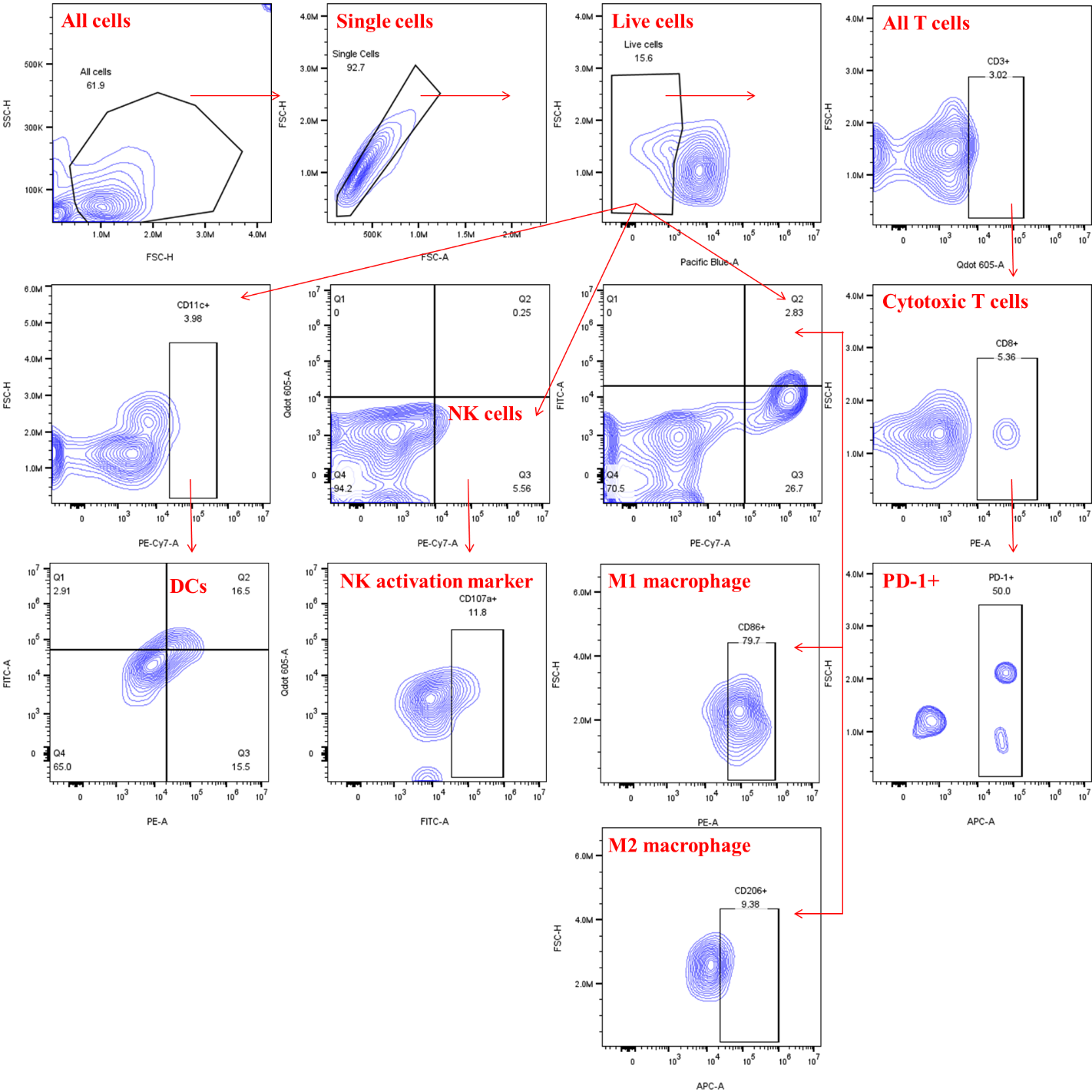


**Fig. 4.** Gating strategy for flow cytometry analysis of immune cells.

**
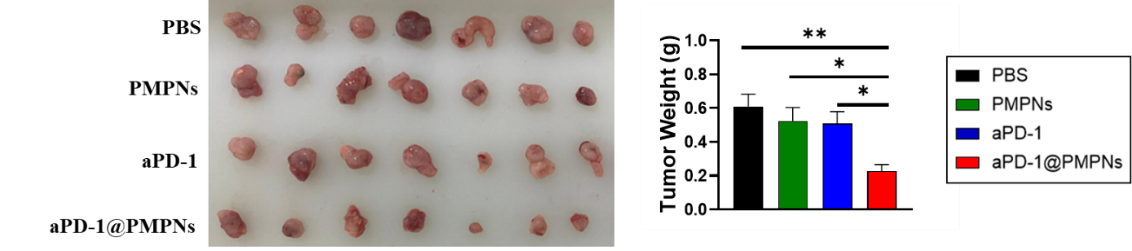
**

**Fig. 5.** The images of excised tumors and corresponding tumor weights.


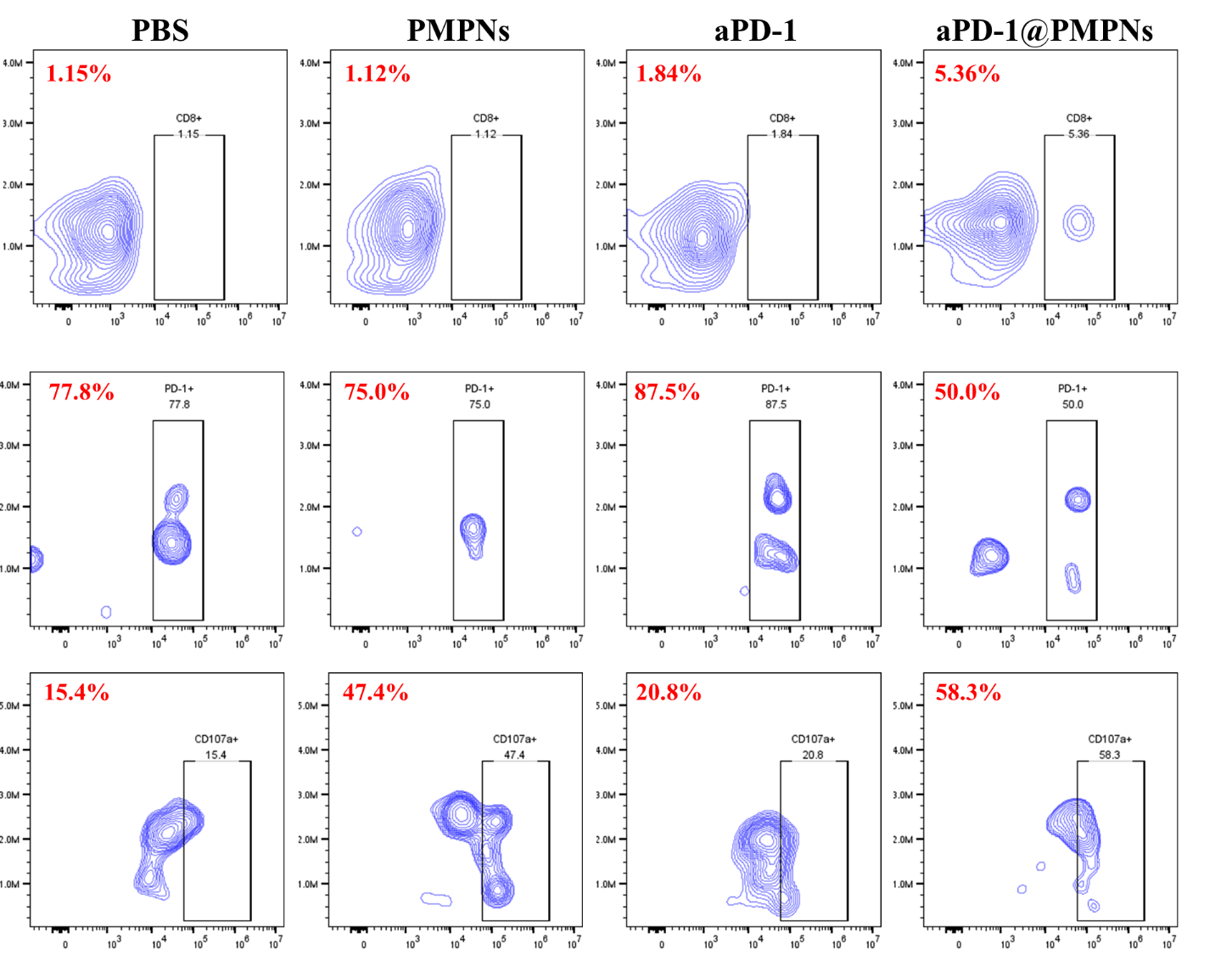


**Fig. 6.** Flow cytometry analysis of CD8+ T cell (CD3+CD8+ as the marker), and PD-1+ (CD3+CD8+PD-1+ as the marker) and CD107a+ (CD3+CD8+CD107+ as the marker) CD8+ T cell populations in the tumor tissue after different treatments.


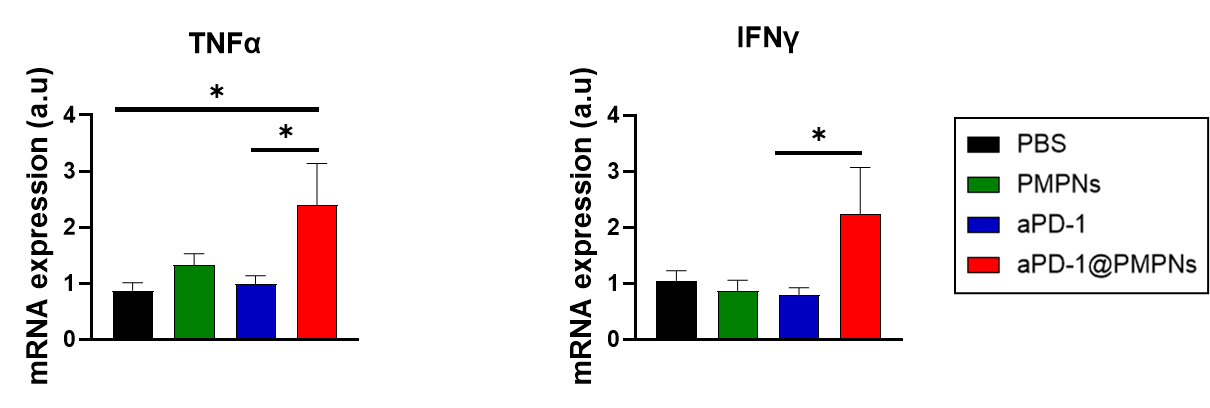


**Fig. 7.** mRNA expression levels of TNFα and IFN-γ in tumor tissues.


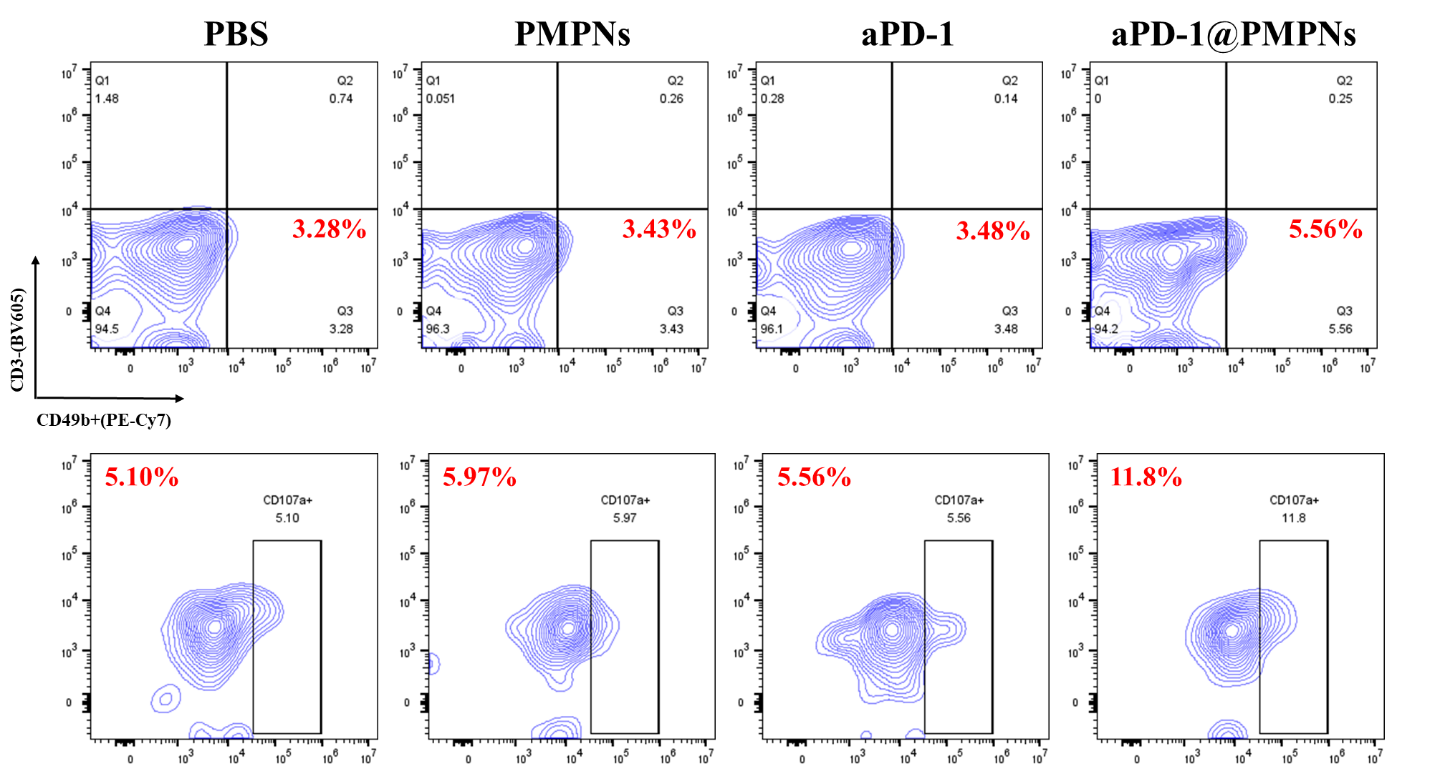


**Fig. 8.** Flow cytometry analysis of NK (CD3-CD49b+ as the marker) and CD107a+ NK (CD3-CD49b+CD107+ as the marker) cell populations in the tumor tissue after different treatments.


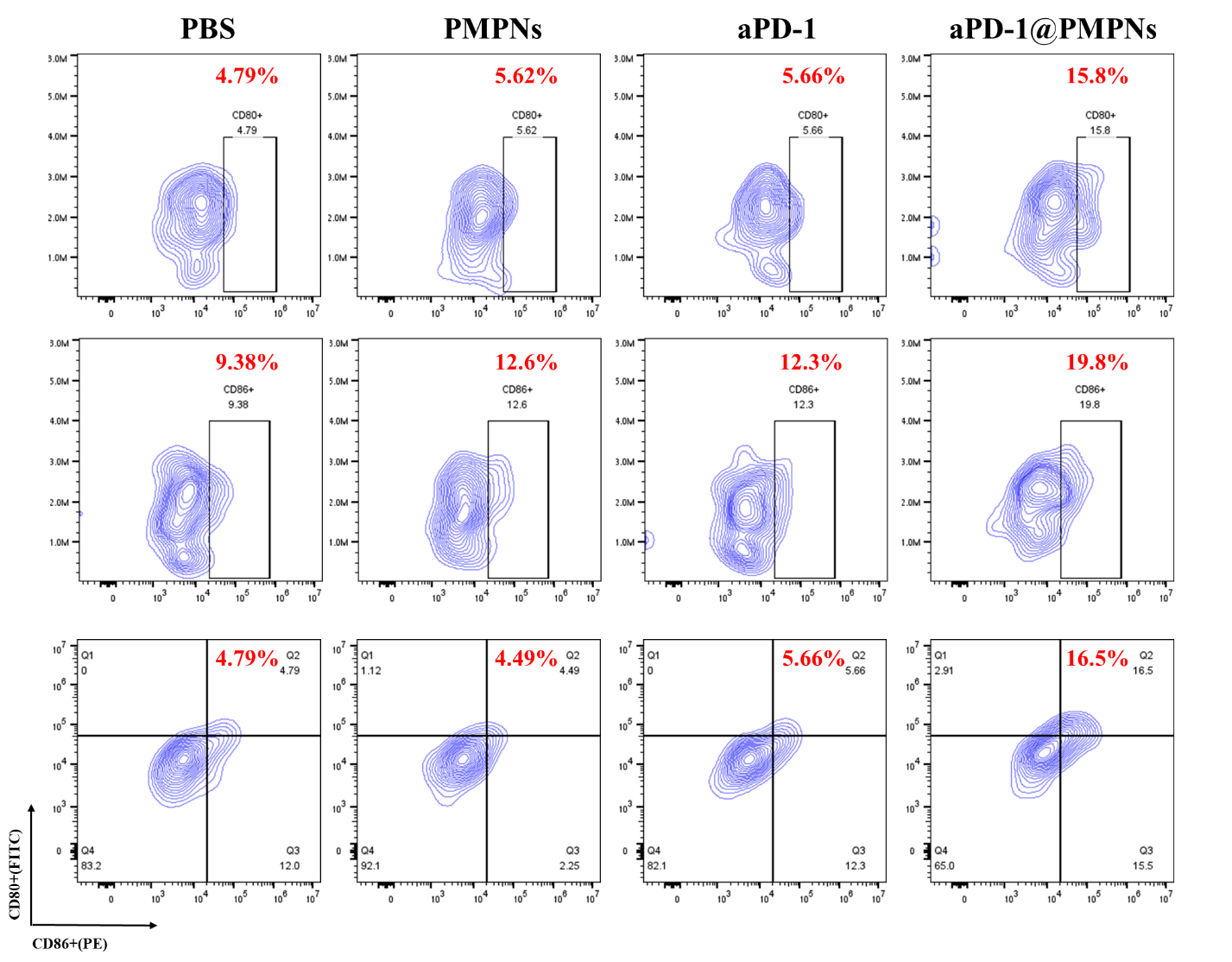


**Fig. 9.** Flow cytometry analysis of CD80+ (CD11c+CD80+ as the marker), CD86+ (CD11c+ CD86+ as the marker), and CD80+CD86+ (CD11c+CD80+CD86+ as the marker) DC populations in the tumor tissue after different treatments.


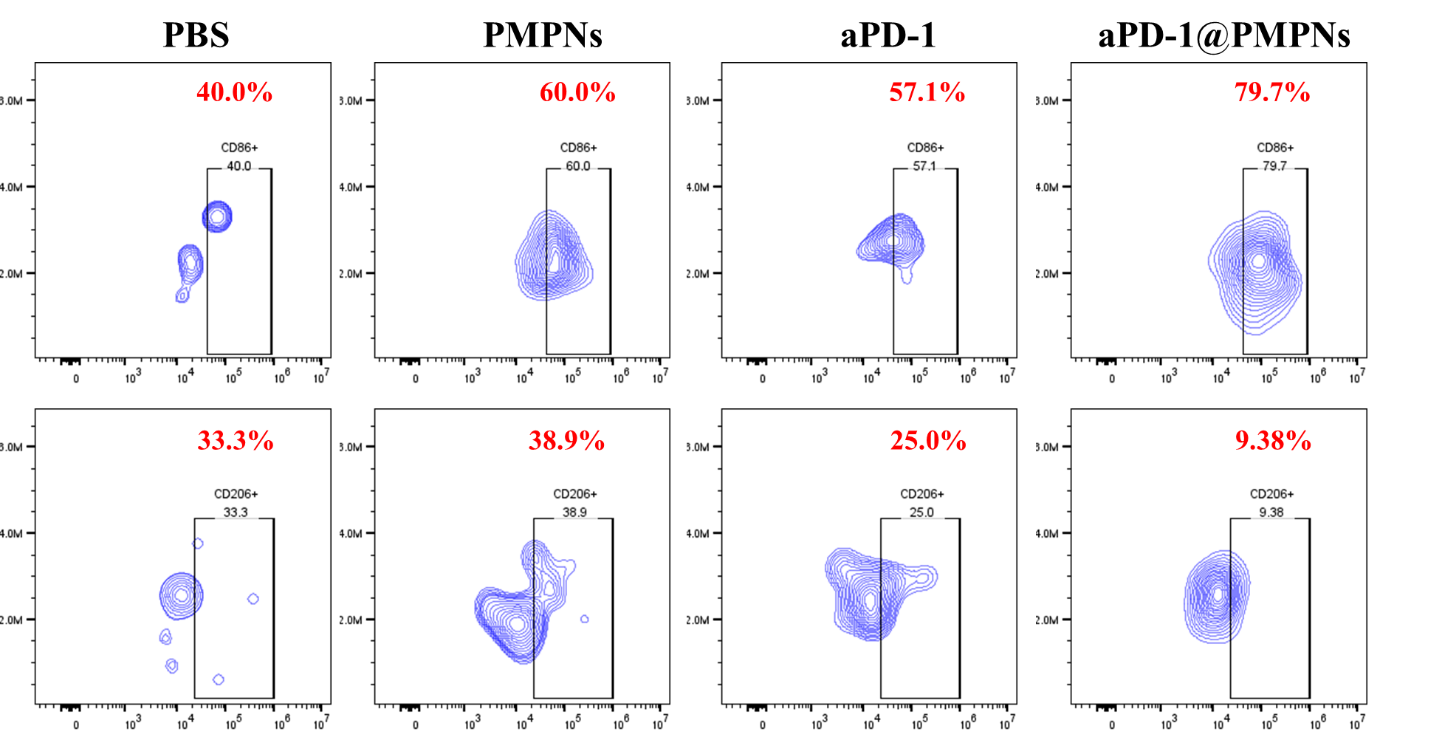


**Fig. 10.** Flow cytometry analysis of M1 (CD11b+F4/80+CD86+ as the marker) and M2 (CD11b+F4/80+CD206+ as the marker) macrophage populations in the tumor tissue after different treatments.


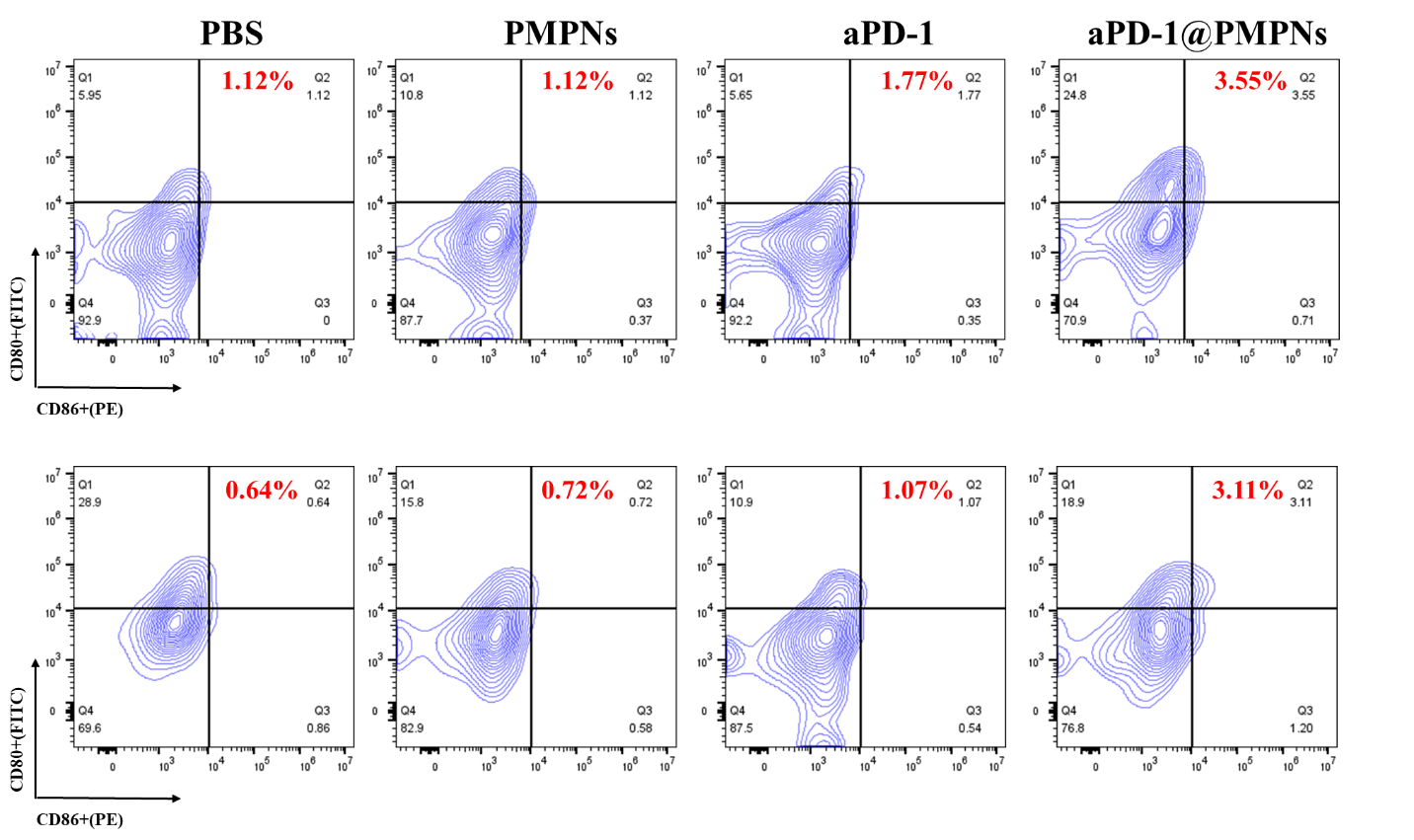


**Fig. 11.** Flow cytometry analysis of CD80+CD86+ DC (CD11c+CD80+CD86+ as the marker) populations in the inguinal and contralateral lymph nodes after different treatments.


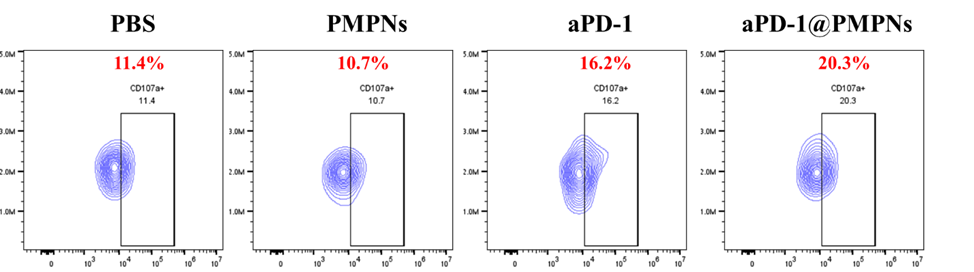


**Fig. 12.** Flow cytometry analysis of CD107a+ CD8+ T cell (CD3+CD8+CD107+ as the marker) populations in the spleen after different treatments.


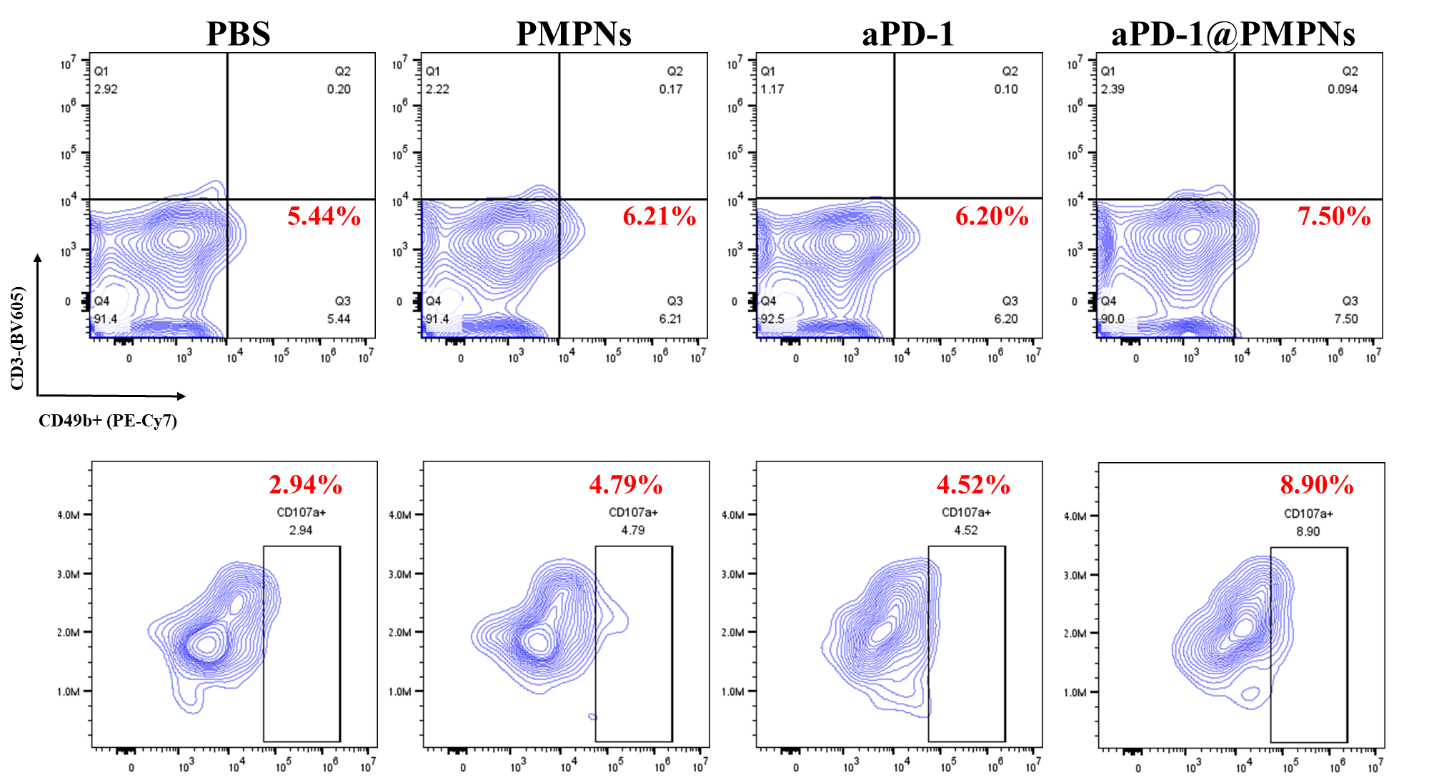


**Fig. 13.** Flow cytometry analysis of NK (CD3-CD49b+ as the marker) and CD107a+ NK (CD3-CD49b+CD107+ as the marker) cell populations in the spleen after different treatments.


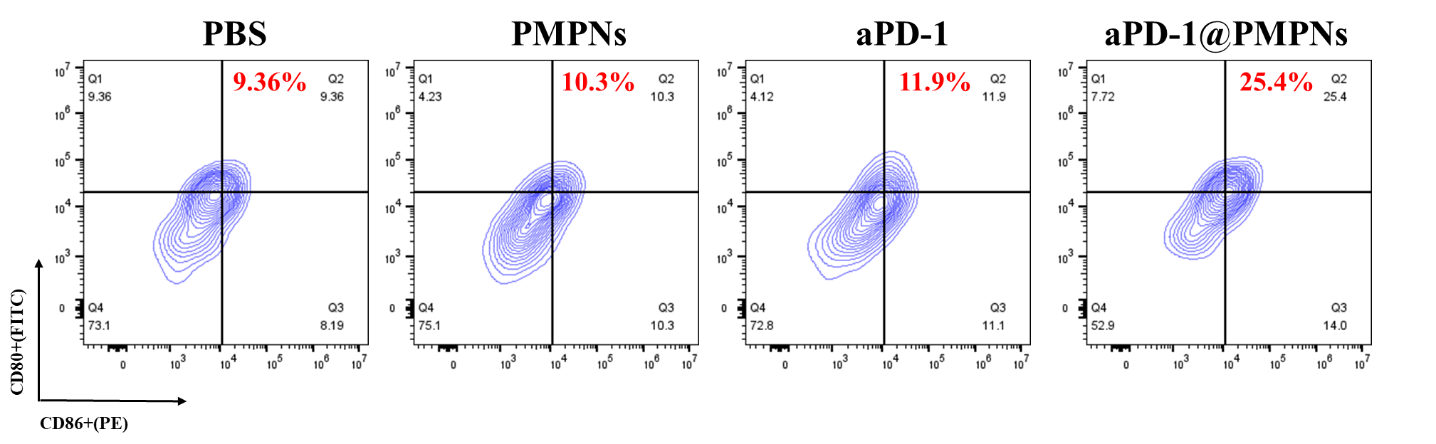


**Fig. 14.** Flow cytometry analysis of CD80+CD86+ DC (CD11c+CD80+CD86+ as the marker) populations in the spleen after different treatments.


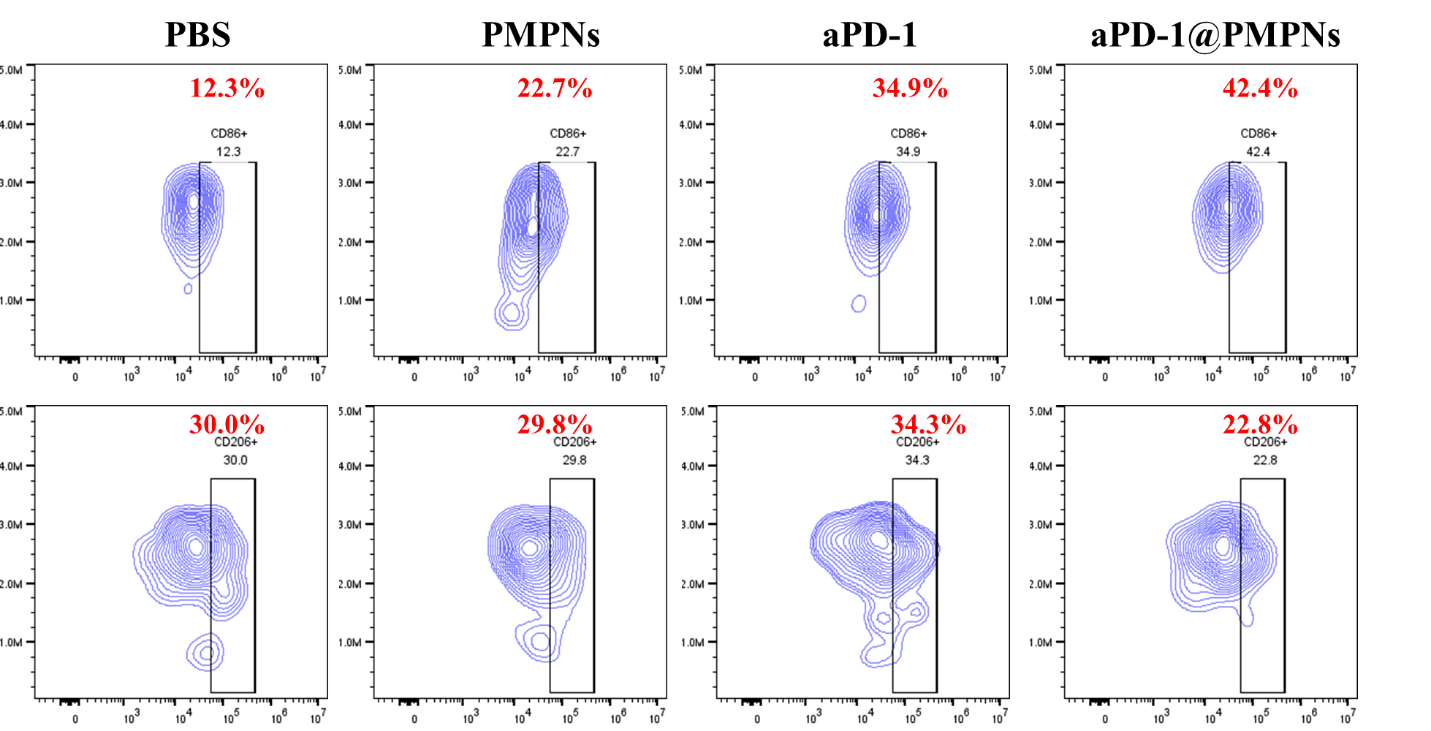


**Fig. 15.** Flow cytometry analysis of M1 (CD11b+F4/80+CD86+ as the marker) and M2 (CD11b+F4/80+CD206+ as the marker) macrophage populations in the spleen after different treatments.

**Table 1: List of antibodies used for flow cytometry**

| **Antibodies** | **Catalogue** | **Clone** |
| --- | --- | --- |
| Fixable Viability Dye eFluor™ 450 | 65-0863-18 | - |
| PD-1 (APC) | 17-9985-82 | J43 |
| PD-L1 | 14-5982-82 | MIH5 |
| CD3e (Brilliant Violet™ 605) | 406-0031-82 | 145-2C11 |
| CD8a (PE) | 12-0081-82 | 53-6.7 |
| CD49b (PE-Cy7) | 25-5971-81 | DX5 |
| CD107a (Alexa Fluor 488) | 53-1071-82 | 1D4B |
| CD80 (FITC) | 11-0801-86 | 16-10A1 |
| CD86 (PE) | 12-0862-82 | GL1 |
| CD206 (APC) | 17-2061-82 | MR6F3 |

**Table 2: List of primer sequences used for qPCR**

| **Primer** | **Sequences** |
| --- | --- |
| IFN-γ | Forward: 5′-GCGTCATTGAATCACACCTG-3′  Reverse: 5′-TGAGCTCATTGAATGCTTGG-3′ |
| TNF-α | Forward: 5′-CGTCAGCCGATTTGCTATCT-3′  Reverse: 5′-CGGACTCCGCAAAGTCTAAG-3′ |
| 18S | Forward: 5′-CGAAAGCATTTGCCAAGAAT-3′  Reverse: 5′-AGTCGGCATCGTTTATGGTC-3′ |

**Table 3: List of antibodies used for western blot analysis**

| **Antibody** | **Catalogue** | **Company** |
| --- | --- | --- |
| Akt (pan) (C67E7) Rabbit mAb | 4691T | Cell Signaling Technology |
| Anti-Phospho-Akt (Ser473) Rabbit Polyclonal Antibody | 9271T | Cell Signaling Technology |
| P44/42 MAPK (Erk1/2) (137F5) Rabbit mAb | 4695T | Cell Signaling Technology |
| XP® Anti-Phospho-p44/42 MAPK (Erk1/2) (Thr202/Tyr204) Rabbit Monoclonal Antibody | 4370T | Cell Signaling Technology |
